# Chicago Medical Society

**Published:** 1867-12

**Authors:** 


### CHICAGO MEDICAL SOCIETY.

### DANGEROUS EFFECTS OF CHLOROFORM.

Dr. Holmes reported a case in which the administration of chloroform was followed by exceedingly alarming symptoms.

The patient, aged 68 years, although he had formerly been addicted to the excessive use of alcohol, was apparently in good health. The anæsthetic was given for performing iridectomy, at the regular clinic at the Chicago Charitable Eye and Ear Infirmary. He was placed in a recumbent position, the head being slightly elevated. The chloroform was inhaled from a napkin, folded square and held, in the form of an arch, a couple of inches from the face. In about four minutes, without any unusual change in the patient's appearance, or in the action of the heart and lungs, there were very violent tonic spasms of the legs and arms. At the end of another minute, the muscles became suddenly relaxed; the breathing ceased, and the pulse became very feeble; the face was covered with perspiration; the lips were pallid, and the features "pinched," giving the appearance of death itself. On raising the feet and hips, drawing out the tongue, and pressing upon the chest, the patient began to breathe. The breathing became normal in two or three minutes, when the patient was restored to a horizontal position. Without giving more chloroform, the breathing at once ceased. The same expedients were immediately adopted, but for some time without effect. Froth stood about the lips, unmoved by the breath; the pulse at the wrist was almost inappreciable. After ten minutes, the patient being held in the position described, and the chest being alternately compressed and relaxed, the heart's action, which had, however, not become entirely inaudible, became stronger, and soon the respiration and pulse became normal. The operation was then performed without further incident. In twenty minutes, the patient was able to walk up two flights of stairs without difficulty.

The reporter stated that he had recently observed as alarming, though not so protracted, symptoms while administering sulph. ether to a patient advanced in years. The reporter also stated his belief, that the importance of position in these cases was not sufficiently impressed upon the minds of practitioners. It was not sufficient, simply to remove the pillow and slightly elevate the hips; the patient should be placed head downward, upon a very steep inclined plane. He had observed the immediate good results of this position in several cases in which chloroform acted unpleasantly upon the heart and lungs.

Whatever may be the conditions, upon which danger in the administration of chloroform depends, there is reason for believing, in some cases at least, that there is an insufficient supply of blood in the brain.

### CHRONIC ASTHMA RELIEVED BY ELECTRICITY.

Dr. Bevan reported a case of most distressing chronic asthma, in a female, 35 years of age, relieved by weak currents of electricity.

### RUPTURE OF THE SPLEEN.

Dr. Fenn reported a case of rupture of the spleen, in a patient 23 years of age, at the County Hospital. The patient had suffered, some time previous, from fever and ague, and recently, from exposure to cold. During the first week at the hospital, the chief symptom was a high fever. During the succeeding two weeks, there were distinct evidences of peritonitis, which terminated in death. At the autopsy, there was found in the abdomen, a coagulum weighing seven pounds. The substance of the spleen presented the appearance of a soft jell.

### SINGULAR FRACTURE OF THE OS CALCIS.

Dr. Bogue exhibited the os calcis of a patient, who had fallen from the second story of a dwelling-house to the sidewalk. The bone was fractured into twelve pieces, no other bone being broken.

### RETAINED PLACENTA.

Dr. Paoli reported a case, in which he was unable, with the greatest effort, to remove but a-third of the placenta. In six days, without any unfavorable symptoms, the remainder of the placenta was expelled.

### STRICTURE.

Dr. Rush reported a case of stricture, in a patient 53 years of age, the result of gonorrhœa contracted twelve years before. For six years, there had been but very little obstruction; for four years subsequently, the patient had experienced much difficulty in voiding the urine, and during the remaining two years, the discomfort had been extreme. At the time of the operation, there were three fistulæ, with quite extensive sinuses—one in the perineum, one in front of the scrotum, and another in the penis. The stricture, about an inch in length, was situated near the middle of the penis. A No. 2 catheter could be passed with difficulty. Forcible dilatation was accomplished by means of a dilator, with scarcely any hemorrhage. In a short time, the patient was well, and able to pass a No. 12 sound without assistance.

### ENLARGED CANCEROUS LIVER.

Dr. Ross exhibited the liver of a patient, who had died at the County Hospital, under somewhat singular circumstances. The patient, a German, 41 years of age, had suffered for eight months from indigestion, and pain in the epigastric region, which he attributed to the poor diet he received on board ship, during a long voyage. A very large swelling in the region of the liver was developed *during the last two weeks of life.* At the autopsy, the liver was found enormously enlarged, weighing fifteen pounds. It was studded with encephaloid masses, varying in size from that of a pea to that of a hen's egg. No other organ presented symptoms of the disease.

### DEATH FROM LARGE DOSES OF DIGITALIS.

Dr. Dunn reported a case, in which a woman, laboring under delirium tremens, died, on suddenly rising, after taking four teaspoonfuls of tinc. digitalis, at intervals of about four hours. Two teaspoonfuls had been prescribed, the other two having been administered by the carelessness of the patient's friends.

Drs. Davis and Fisher reported cases of delirium tremens, under the care of these physicians, which had terminated very suddenly, after large and repeated doses of tinct. digitalis had been taken.

### BENDING OF THE RADIUS AND ULNA.

Dr. Reed reported a case, in which the fore-arm of a child, 5 years of age, had been bent, by a fall, almost to an acute angle, without any apparent fracture.

### PERFORATING ULCER OF THE ILEUM.

Dr. Ross exhibited the ileum of a young man, who died suddenly during convalescence from typhoid fever. The specimen showed a small perforation into the peritoneal cavity.

### FACIAL PARALYSIS IN SYPHILIS.

Dr. C. G. Smith reported a case of facial paralysis, as a symptom of secondary syphilis, six weeks after the primary disease. This entirely relieved by the use of protiodide of mercury. Such cases are regarded as quite rare.

### TETRACHLORIDE OF CARBON.

Prof. Andrews described the very alarming symptoms which followed the inhalation of the tetrachloride of carbon. The patient, a young man, somewhat reduced by confinement and disease, after breathing this anæsthetic some minutes, complained of violent griping pain in the abdomen. The pulse increased very rapidly in frequency, till it could scarcely be counted; respiration ceased; the head was drawn back; and the pupil dilated, after about ℥iij. were inhaled. The usual efforts to arouse the patient were made, with success. In a few minutes, the patient was completely out of danger.

Dr. Marguerat reported a case of mania following erysipelas, evidently produced by suppression of urine (uræmia). The renal secretion was soon reëstablished, after the administration of turpentine and spirits of nitre, when the delirium disappeared.

### BROMIDE OF POTASH IN EPILEPSY.

Dr. Merryman related a case of severe epilepsy, much ameliorated by the use of bromide of potash.

### VALVULAR DISEASE OF THE HEART.

Dr. Ross exhibited the heart of a male patient, 38 years of age, who had suffered a severe attack of acute rheumatism fourteen years ago. The heart was hypertrophied, the mitral valves much thickened and indurated, the aperture being reduced to a very short and narrow slit, and the aortic valves were also indurated and thickened.

